# Preparation and Characterization of Polycarbonate-Based Blend System with Favorable Mechanical Properties and 3D Printing Performance

**DOI:** 10.3390/polym15204066

**Published:** 2023-10-12

**Authors:** Hao Liu, Simin Chen, Chengdi Li, Xiao Chen, Jinbo Li, Ping Chen, Fuzhen Xie, Huihua Jian, Xiaoying Huang, Lei Liu

**Affiliations:** Xinyu Key Laboratory of Materials Technology and Application for Intelligent Manufacturing, School of Mechanical and Electrical Engineering, Xinyu University, Xinyu 338004, China

**Keywords:** material extrusion, polycarbonate, polymer blending, mechanical properties, 3D printing performance

## Abstract

Recently, material extrusion (MEX) 3D printing technology has attracted extensive attention. However, some high-performance thermoplastic polymer resins, such as polycarbonate (PC), cannot be processed by conventional MEX printing equipment due to poor processing performance. In order to develop new PC-based printing materials suitable for MEX, PC/poly(butylene adipate-co-terephthalate) (PBAT) blends were prepared using a simple polymer blending technique. It was found that the addition of PBAT component significantly improved processing performance of the PC, making the blends processable at 250 °C. More importantly, the PC was completely compatible with the PBAT, and the PBAT effectively reduced the T_g_ of the blends, endowing the blends with essential 3D printing performance. Furthermore, methyl methacrylate-butadiene-styrene terpolymer (MBS) was introduced into the PC/PBAT blends to improve toughness. SEM observations demonstrated that MBS particles, as stress concentration points, triggered shear yielding of polymer matrix and absorbed impact energy substantially. In addition, the MBS had little effect on the 3D printing performance of the blends. Thus, a PC/PBAT/MBS blend system with favorable comprehensive mechanical properties and 3D printing performance was achieved. This work can provide guidance for the development of novel MEX printing materials and is of great significance for expanding the variety of MEX printing materials.

## 1. Introduction

Polycarbonate (PC) is an amorphous ester polymer containing carbonate groups in molecular chains. Due to the presence of benzene rings and quaternary carbon atoms in main chains, the PC has excellent mechanical strength and heat resistance; ether single bonds give the molecular chains a certain flexibility, and the PC exhibits good toughness at room temperature. In addition, steric hindrance caused by the rigid molecular chains and four-substituted quaternary carbon atoms makes the PC difficult to crystallize, and therefore the PC has high transparency. Overall, the PC is a versatile engineering plastic with excellent comprehensive performance, and is the only plastic with well transparency among engineering plastics, which is widely used in electronic appliances [1], automobiles [2], tissue scaffolds [3], building materials [4], and other fields [5].

The PC is a thermoplastic polymer resin, and its common processing methods include extrusion, injection molding, hot pressing, etc. However, due to harsh processing conditions, such as high melting temperature and glass transition temperature, the PC is not suitable for material extrusion (MEX) 3D printing technology. Due to unique advantages in complex shape forming and personalized customization, 3D printing technology has important application prospects in various fields, including aerospace [6], maritime [7], and biomedical [8]. Among the 3D printing technologies, the MEX is the most widely used method because of its simple operation and low equipment cost [9,10]. The development of new printing materials is an important research direction in MEX technology [11,12]. However, this technology puts forward the following requirements for MEX printing performance of the materials: (1) suitable processing flowability ensures that printing filaments can be smoothly extruded from the nozzle; (2) appropriate softening temperature guarantees that the filaments can be deposited and adhered to the printing platform; (3) well strength and toughness prevent the filaments from bending or breaking during the printing process; (4) good dimensional stability avoids warping and shrinkage of 3D printed products.

At present, commercial MEX printing consumables mainly include acrylonitrile-butadiene-styrene copolymer (ABS) [13], polylactic acid (PLA) [14], and so on. Most single-component thermoplastic polymer resin materials still have problems when used as MEX printing consumables [15,16,17]. Therefore, many scholars are committed to developing new MEX printing materials [18,19]. Among them, polymer blending is an economical and effective method that can overcome the shortcomings of single-component polymers, make the performance of polymer blends more balanced, and meet the needs of practical applications at lower production costs. Jia et al. prepared PA6/POE-g-MAH/PS blends by adding random maleic anhydride grafted ethylene octene copolymer (POE-g-MAH) and polystyrene (PS) into polyamide 6 (PA6) matrix [20]. It was claimed that due to the decline in crystallinity of the PA6 matrix, the internal stress of 3D printed products decreased significantly, solving the warping problem of the PA6 during MEX printing. Similarly, Peng et al. prepared PP/POE-g-MAH/PA6 blends to overcome the shrinkage phenomenon of polypropylene (PP)-based 3D printed products [21]. Spoerk et al. prepared PP-based composites by introducing PP-g-MAH and spherical expanded perlite. The results indicated that the shrinkage rate of the composite containing 30 vol% fillers decreased by 34%, and the warpage degree of corresponding 3D printed products was also reduced [22]. Taking these as inspiration, the processing performance of the PC could be improved through polymer blending technology to meet the requirements of the MEX process. At present, PC-based polymer blends have been reported, such as PC/ABS [23,24], PC/PLA [25] blends, etc. The research on PC-based blends has mainly focused on toughening [26,27], compatibilization [28,29], and transesterification [30,31], but the application of PC-based blends in MEX technology has rarely been reported.

Poly(butylene adipate-co-terephthalate) (PBAT) is a thermoplastic semi-crystalline polyester, which contains aliphatic polyester and aromatic polyester in molecular chains, so the PBAT possesses good processing fluidity and heat resistance. In this work, PC/PBAT blends with different PBAT contents were prepared by introducing a PBAT component into PC matrix. The processing, mechanical, thermal properties, compatibility, and microstructure of the PC/PBAT blends were systematically investigated. Furthermore, methyl methacrylate-butadiene-styrene terpolymer (MBS) was used as a toughening agent to improve the toughness of the PC/PBAT blends, the influence of the MBS on mechanical properties of the blend system was studied, and the toughening mechanism was elucidated. On this basis, the PC-based blend system was used to fabricate 3D printed products via MEX, and the 3D printing performance of this blend system was evaluated. The purpose of this work was to develop a PC-based blend system with favorable mechanical properties and 3D printing performance through simple polymer blending technology, which not only broadened the types of MEX printable materials, but also provided a reference for the expansion of MEX available materials.

## 2. Experimental

### 2.1. Materials

Polycarbonate (PC) pellets (WONDERLITE^®^ PC-110) with a melt volume rate of 10 mL/10 min (300 °C, 1.2 kg) and a specific gravity of 1.2 g/cm^3^ were purchased from CHIMEI Corporation (Zhangzhou, China). Poly(butylene adipate-co-terephthalate) (PBAT) pellets (ecoflex^®^ C1200) with a melt flow rate of 2.7–4.9 g/10 min (190 °C, 2.16 kg) and a specific gravity of 1.25–1.27 g/cm^3^ were purchased from BASF SE. Methyl methacrylate-butadiene-styrene terpolymer (MBS) powders (PARALOID™ EXL-2620) with a specific gravity of 0.35–0.45 g/cm^3^ were purchased from Dow Inc., Midland, MI, USA.

### 2.2. Preparation of PC/PBAT and PC/PBAT/MBS Blends and Corresponding MEX Filaments

First, PC pellets were dried at 100 °C for 8 h to remove excess moisture, and PBAT pellets and MBS powders were dried at 60 °C for 8 h. According to the formulations in Table 1 and Table 2, the raw materials were placed in plastic bags in proportion and mixed evenly. Then, polymer blending was performed using a twin-screw extruder (Polylab OS 14/40, Haake, Germany), and resulting extrudates were granulated to obtain PC/PBTA and PC/PBTA/MBS blends. The extrusion temperature range was set at 240–265 °C, and the extrusion speed was 60 r/min. Finally, the obtained blends were dried and then processed via the twin-screw extruder to prepare MEX filaments with a diameter of about 1.75 mm under the same process parameters.

### 2.3. Preparation of 3D Printed Specimens

The 3D model files (STL format) of specimens were imported into a slicing software (Cura 15.04 version). After adjusting printing parameters and model position, G-code that could be recognized by a MEX printer (S1 Architect 3D, Guoguang Instruments, Shanghai, China) was exported. The as-prepared MEX filaments were driven into print nozzle by stepping gear, and the printer automatically recognized the G-code and fabricated the specimens according to an established route. The printing parameters used are shown in Table 3.

### 2.4. Preparation of Specimens by Injection Molding

For comparison, the above blends were processed via an injection molding machine (P Series 50 e, ONLY, Foshan, China) to prepare standard specimens for testing mechanical properties. The processing temperature range was 240–265 °C. Injection pressure, injection rate, packing pressure, back pressure, mold temperature, and cooling time were set to 40 MPa, 40 mm/s, 20 MPa, 3 MPa, 25 °C, and 18 s, respectively. After the injection molding was completed, the specimens were placed at room temperature for 24 h to eliminate internal stress before testing.

### 2.5. Characterization

#### 2.5.1. Torque Rheological Test

The raw materials were mixed according to the formulations, and then torque rheological testing was performed using a torque rheometer (RTOI-55/20, POTOP, Guangzhou, China). Test temperature, rotational speed, and test time were 250 °C, 50 r/min, and 5 min, respectively.

#### 2.5.2. Capillary Rheological Test

A capillary rheological test was performed at 240 °C via a capillary rheometer (RHEOLGIC 5000, CEAST, Pianezza, Italy). The capillary diameter was 1 mm, and the length–diameter ratio was 30/1. The shear rates were 50, 100, 200, 500, 800, 1000, and 2000 s^−1^.

#### 2.5.3. Mechanical Test

A tensile test and a flexural test were conducted on a universal mechanical testing machine (Z010, Zwick/Roell, Ulm, Germany) at a test rate of 10 mm/min at ambient temperature. The tensile test referred to the standard ISO 527-2-2012 (type 1BA) [32], and the flexural test referred to the standard ISO 178-2010 (80 × 10 × 4 mm) [33]. The impact performance was measured using a cantilever beam impact testing machine (5113, Zwick/Roell, Ulm, Germany) with a 5.5 J pendulum. The impact test referred to the standard GB/T 1843-2008 (63.5 × 12.7 × 4 mm, A-notch) [34].

#### 2.5.4. Differential Scanning Calorimetry (DSC)

Crystallization and melting behavior were analyzed using a differential scanning calorimeter (DSC 204F1, NETZSCH, Waldkraiburg, Germany) under N_2_ atmosphere. First, the thermal history was eliminated by increasing the temperature from room temperature to 250 °C at a rate of 20 °C/min and maintaining at 250 °C for 3 min. Then, the temperature was reduced to 20 °C at a rate of 10 °C/min and kept at 20 °C for 5 min. The data during the cooling process were collected as crystallization curves. Finally, the temperature was increased again to 250 °C at a rate of 10 °C/min, and the data during the heating process were collected as melting curves.

#### 2.5.5. Thermal Gravimetric Analysis (TGA)

Thermal stability was analyzed using a thermogravimetric analyzer (TG 209 F1, NETZSCH, Selb, Germany) under N_2_ atmosphere at a heating rate of 20 °C/min.

#### 2.5.6. Dynamic Mechanical Analysis (DMA)

Dynamic mechanical performance was measured using a dynamic mechanical analyzer (Q800, TA Instruments, New Castle, DE, USA) at a heating rate of 3 °C/min, a frequency of 1 Hz, and a strain of 0.05%.

#### 2.5.7. Vicat Softening Temperature (VST)

Heat resistance was analyzed using a Vicat softening point tester (HDT 3 VICAT, CEAST, Turin, Italy) in accordance with the standard GB/T 1633-2000 [35]. The heating rate was 120 °C/h, and the load on the sample was 50 N. Three samples were measured in each group, and the average value was taken as the VST, provided that the difference between the measured values in the same group did not exceed 2 °C.

#### 2.5.8. Scanning Electron Microscope (SEM)

Morphology was observed with a field-emission SEM (Nano 430, FEI, Eindhoven, The Netherlands) at an acceleration voltage of 10 kV in ambient temperature. Before observation, the samples were cryogenically fractured in liquid nitrogen and then treated with gold spray.

## 3. Results and Discussion

### 3.1. Effect of PBAT on Processing Performance of PC/PBAT Blends

#### 3.1.1. Processing Torque

**Figure 1:**
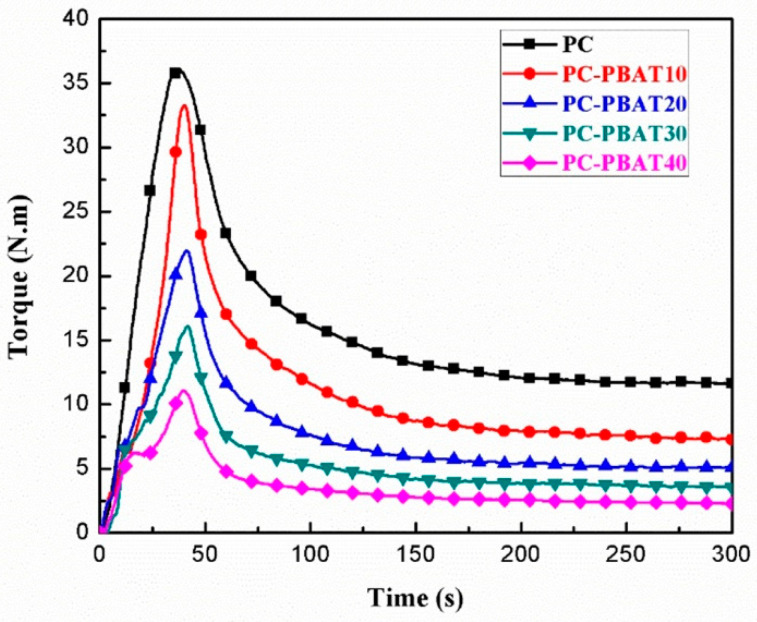
Processing torque curves of the PC/PBAT blends.

The equilibrium torque during processing can be used to characterize fluidity of polymer melt and reflect processing performance of polymer. The greater the equilibrium torque, the worse the processability of the polymer melt at this temperature. As a common engineering plastic, PC has a processing temperature in the range of 260–300 °C. The prerequisite for applying the PC to conventional MEX technology is to ensure that the PC/PBAT blends have favorable processability at temperatures around 250 °C (maximum temperature of the nozzle of the MEX printer). Figure 1 shows the influence of PBAT content on processing torque of the PC/PBAT blends at a processing temperature of 250 °C. It can be seen that the processing torque of each component increased abruptly first and then decreased until equilibrium, corresponding to the process of polymer transformation from solid to melt. As shown in Table 4, the addition of the PBAT significantly reduced the equilibrium torque of the PC. For example, when the PBAT content was 30%, the equilibrium torque of the blend decreased to 3.5 N·m, which was 70.1% lower than that of pure PC, indicating that the PBAT component effectively improved the processing performance of the PC at 250 °C. On the other hand, maximum torque peak also showed a large decline with the addition of the PBAT component, which was beneficial to saving the energy consumption of processing equipment.

#### 3.1.2. Capillary Rheological Properties

**Figure 2:**
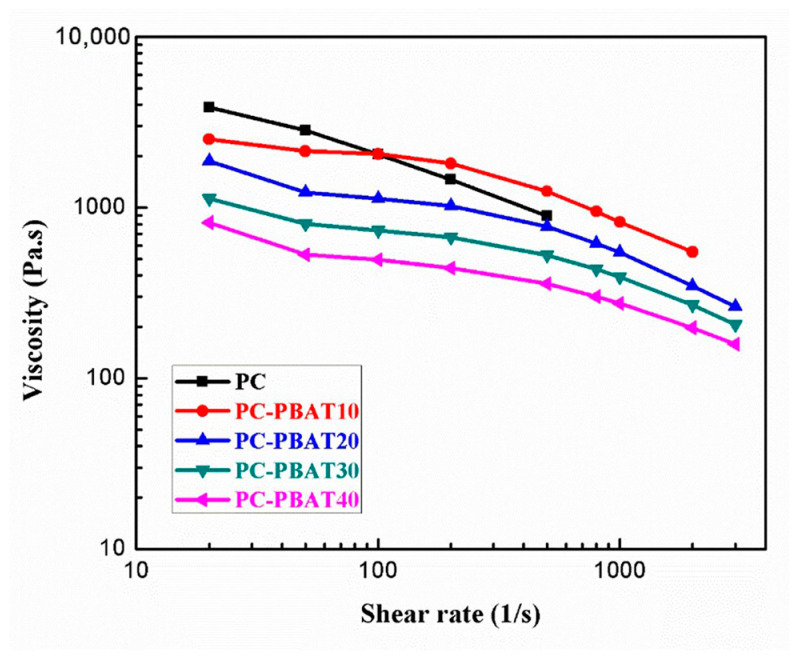
Viscosity–shear rate curves of the PC/PBAT blends.

In addition to the processing torque, the capillary rheological properties of the PC/PBAT blends were also investigated. Capillary rheological behavior can more directly reflect the processability of polymer by measuring the shear stress of polymer melt at different shear rates, and can simulate melt flow during extrusion and MEX 3D printing. Different processing methods correspond to different shear rates. For example, shear rate for extrusion molding is within the range of 10^−2^–10^3^ s^−1^, while for injection molding it is within the range of 10–10^4^ s^−1^. Therefore, the shear rate range for this experiment was set at 20–3000 s^−1^. The effect of the PBAT component on rheological properties of the PC/PBAT blends at 240 °C is shown in Figure 2. It was found that the viscosity of the blends decreased as the shear rate increased, showing typical shear thinning characteristics of non-Newtonian fluid. More importantly, the viscosity of the blends decreased markedly with increasing PBAT content. At lower shear rates (below 100 s^−1^), the viscosity of the blends was reduced by an order of magnitude. This was because the PBAT is a linear polyester with a lower melting point, and its introduction dramatically improved the flowability of the PC, making the PC processable at 240 °C.

**Figure 3:**
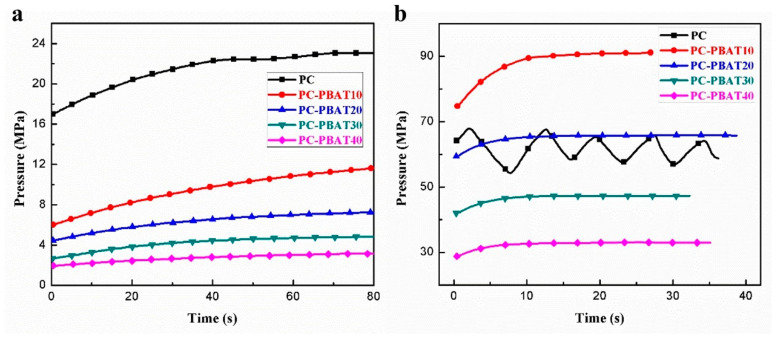
Die pressure–time curves of the PC/PBAT blends at shear rates of (**a**) 50 s^−1^ and (**b**) 1000 s^−1^.

Figure 3 shows the die pressure–time curves of the PC/PBAT blends at two different shear rates. It was found that normal rheological data of pure PC were obtained at a shear rate of 50 s^−1^ (Figure 3a); however, the rheological curve of pure PC was distorted and pressure oscillation appeared when the shear rate rose to 1000 s^−1^ (Figure 3b). The reason for this phenomenon was that pure PC had poor flowability at 240 °C, and the melt of the PC may produce wall slip at a high shear rate, causing fluctuations in die pressure. It was worth noting that capillary extrusion behavior of the PC/PBAT blends returned to normal after adding the PBAT component, and the pressure oscillation phenomenon disappeared. Moreover, the die pressure continuously decreased as the PBAT content increased, which improved processing stability of the PC/PBAT blends and helped to save energy consumption of processing equipment. In conclusion, the addition of the PBAT component can effectively improve the processing performance of the PC, reducing processing temperature of the PC/PBAT blends to below 250 °C, which was a prerequisite for the application of the PC/PBAT blends in MEX technology.

### 3.2. Effect of PBAT on Thermal Properties of PC/PBAT Blends

#### 3.2.1. Melting and Crystallization Behavior

**Figure 4:**
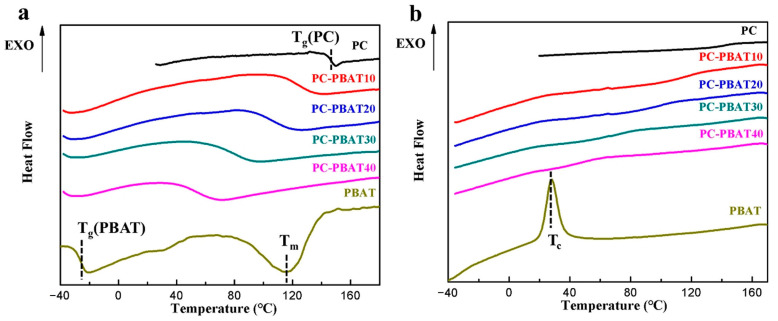
DSC curves measured for the PC/PBAT blends with different PBAT contents: (**a**) heating curves, (**b**) cooling curves.

The glass transition temperature (T_g_) can be used to measure the compatibility of the polymer blend system. Specifically, a compatible blend system has only one T_g_, which is between the T_g_ of each component; a partially compatible blend system has two or more T_g_, but the T_g_ of each component is close to each other; the T_g_ of each component in a completely incompatible blend system remains unchanged. DSC curves measured for the PC/PBAT blends were illustrated in Figure 4, and corresponding DSC data were summarized in Table 5.

As can be seen from Figure 4a, pure PBAT was a semi-crystalline polymer with a T_g_ of −26.3 °C, while pure PC showed a T_g_ of 147.0 °C, much higher than that of pure PBAT. Notably, the PC/PBAT blend system had only one T_g_, and the T_g_ of the blends showed a downward trend with the increase of PBAT content. In addition, the FOX equation was employed to predict T_g_ of copolymerization or compatible blend system [36]. As shown in Figure S1, the T_g_ of the PC/PBAT blend system basically conformed to the FOX fitting curve, indicating that the PC and PBAT components were indeed compatible. The T_g_ of the PC/PBAT blend system can be tailored by controlling PBAT content according to actual application requirements. It was also found that pure PBAT exhibited obvious cold crystallization behavior during the cooling process (Figure 4b), corresponding to a cold crystallization temperature (T_c_) of 28.2 °C. However, the PBAT component did not crystallize in the PC/PBAT blend system, which may be due to their excellent compatibility. That is to say, the PBAT and PC components achieved compatibility at a molecular chain level, resulting in the inability of the PBAT molecular chains to be arranged regularly.

#### 3.2.2. Dynamic Mechanical Performance

**Figure 5:**
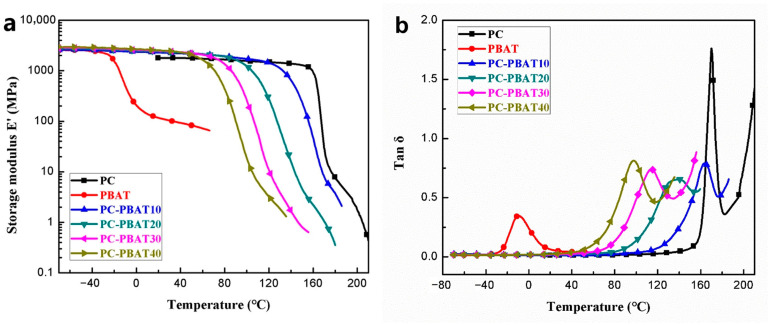
DMA curves measured for the PC/PBAT blends with different PBAT contents: (**a**) storage modulus E’, (**b**) Tan δ.

The DMA curves of the PC/PBAT blends are shown in Figure 5, and corresponding DMA data are summarized in Table 6. Storage modulus E’ reflects the energy storage in polymer materials at a certain frequency of strain, and Tan δ represents the polymer molecular chain mobility. It is well known that T_g_ is a characteristic temperature of polymer chains transition from glassy state to rubbery state, corresponding to Tan δ peak temperature. Therefore, the DMA is also a commonly used method for measuring T_g_. It should be pointed out that the results of DMA and DSC are generally not compared due to the different measurement principles of T_g_ value.

As can be seen from Figure 5a, pure PC was in a glassy state at room temperature with a storage modulus E’ of 1795 MPa, and pure PBAT was in a rubbery state with a storage modulus E’ of 110 MPa. The addition of the PBAT enhanced the E’ of the PC/PBAT blends, and the E’ of the blends increased slightly with increasing PBAT content. This was due to the fact that the rubbery PBAT component that was compatible with the PC at the molecular chain level improved the energy storage capacity of the blends. As shown in Figure 5b, the PC/PBAT blend system showed only one Tan δ peak temperature, and the Tan δ peak temperature of the blends gradually approached that of pure PBAT as the PBAT content increased, which was consistent with the DSC results and further confirmed that the PC and PBAT components were entirely compatible.

#### 3.2.3. Thermal Decomposition Behavior

**Figure 6:**
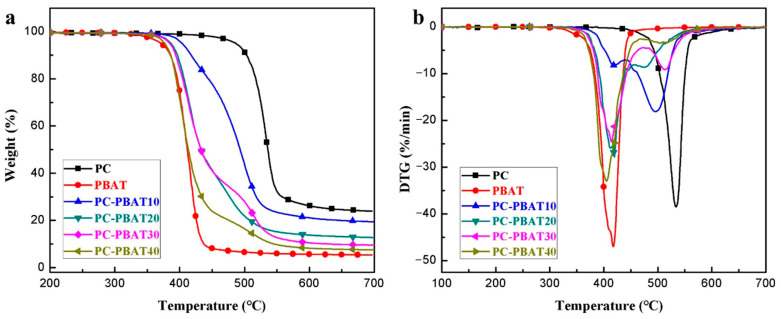
(**a**) TG and (**b**) DTG curves measured for the PC/PBAT blends with different PBAT contents.

The influence of the PBAT component on thermal decomposition behavior of the PC/PBAT blends was studied via TGA. Thermogravimetric (TG) curves and differential thermogravimetric (DTG) curves were shown in Figure 6, and corresponding data were listed in Table 7. The temperature at 5% mass loss in the TG curves was taken as initial decomposition temperature (T_onset_), and the peak temperature in the DTG curves was defined as the maximum thermal decomposition rate temperature (T_max_).

The results indicated that the PC had good thermal stability with a T_onset_ of 486.7 °C and a T_max_ of 533.6 °C, while the PBAT, as a biodegradable polymer, began to decompose at 371.2 °C. The T_onset_ of the PC/PBAT blends decreased with increasing PBAT content and gradually approached that of the PBAT component. In addition, it was observed that there were two peaks in the DTG curves of the blends, corresponding to thermal decomposition processes of the PBAT (T_max1_) and PC (T_max2_) components, respectively. The T_max1_ of the PBAT component in the blends showed little change compared to pure PBAT, while the T_max2_ of the PC component was lower than that of pure PC, indicating that the addition of the PBAT reduced the thermal stability of the PC. Nevertheless, the PC/PBAT blends remained thermally stable without thermal degradation at the processing temperature used in this work. In addition, even if heated to 700 °C in N_2_ atmosphere, the PC could not be completely decomposed due to the presence of benzene rings and carbonate groups, resulting in a lot of residual ash. To sum up, the thermal stability of the PC/PBAT blends was between the PC and PBAT components, and the PBAT content should be maintained within a reasonable range to ensure certain thermal stability.

#### 3.2.4. Heat Resistance

VST is an important indicator for measuring heat resistance of polymer materials. The heat resistant of a polymer generally depends on its T_g_ value. Specifically, as ambient temperature increases, the mobility of polymer chain segments is continuously enhanced, and the chain segments gradually transition from a frozen state to an active state, causing the polymer to soften and deform under load. Therefore, the T_g_ of the polymer is the upper limit of the service temperature. The VST values of the PC/PBAT blends with different PBAT contents are listed in Table 8. It was found that the VST of the blends showed a downward trend with increasing PBAT content. As analyzed above, the VST of the blends was closely related to the T_g_. According to the VST results, the amount of PBAT in the PC/PBAT blend system should not exceed 40%, otherwise the usage temperature would be too low.

### 3.3. Effect of PBAT on Mechanical Properties of PC/PBAT Blends

**Figure 7:**
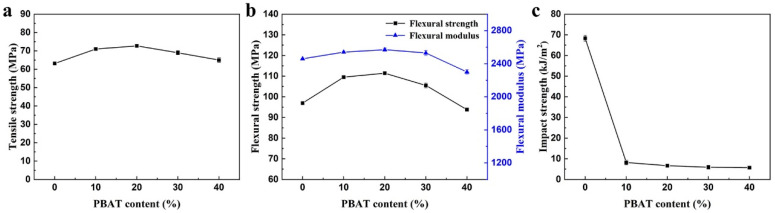
Mechanical properties of the PC/PBAT blends with different PBAT contents: (**a**) tensile strength, (**b**) flexural strength and flexural modulus, (**c**) impact strength.

Mechanical properties of the PC/PBAT blends with different PBAT contents are shown in Figure 7. It was found that tensile strength, flexural strength, and flexural modulus of the blend system presented a trend of a slight increase at first and then a slight decrease with increasing PBAT content. For example, the tensile strength, flexural strength and flexural modulus of the PC-PBAT20 reached 72.7 MPa, 111.4 MPa, and 2570 MPa, respectively, which increased by 15.1%, 14.9%, and 4.5% compared with pure PC. However, the addition of the PBAT seriously deteriorated impact strength of the blends. This was attributed to the fact that the well compatibility of the PC and PBAT components at the molecular chain level broke loose stacking of PC chains, enhanced interaction between molecular chains, and reduced the movable space of chain segments. As a result, the toughness of the PC/PBAT blends witnessed a dramatic decline.

### 3.4. Morphology of PC/PBAT Blends

**Figure 8:**
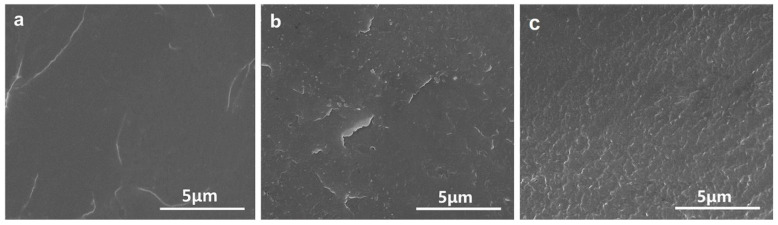
Cryogenically fractured surface morphology of the PC/PBAT blends: (**a**) pure PC, (**b**) PC-PBAT10, (**c**) PC-PBAT30.

As is well known, PC is a kind of polymer material with high light transmittance. After blending with other polymers, the PC usually loses transparency due to differences in the refractive index of the components or crystallization of the components. As shown in Figure S2, the PC/PBAT blends still maintained transparency, indicating that the PC was compatible with the PBAT and no crystallization occurred in any component. Furthermore, the phase structure of the PC/PBAT blends was observed via SEM, as shown in Figure 8. It was found that pure PC exhibited smooth and flat surface, and the PC/PBAT blends showed a single continuous phase. With the addition of the PBAT, the surface of the blends only became slightly rough. From the perspective of microstructure, it was once again confirmed that the PC/PBAT blends had good compatibility.

**Figure 9:**
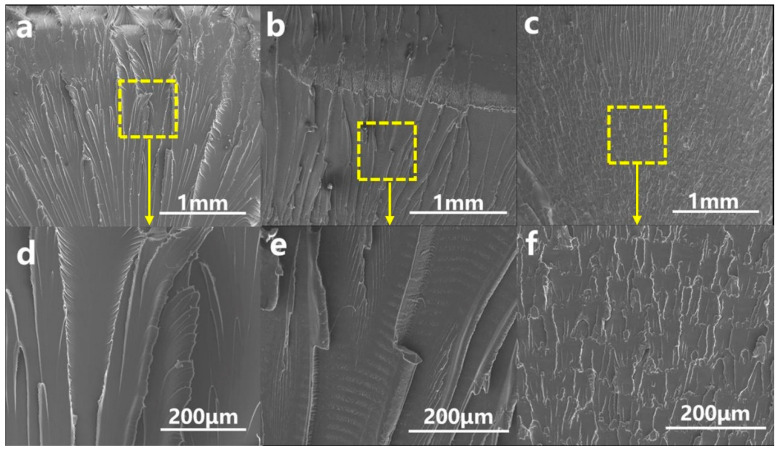
Impact-fractured surface morphology of the PC/PBAT blends: (**a**,**d**) pure PC, (**b**,**e**) PC-PBAT10, (**c**,**f**) PC-PBAT30.

Furthermore, the morphology of the PC/PBAT blends after conducting an impact test was observed. As shown in Figure 9a,d, the impact-fractured surface morphology of pure PC showed apparent shear deformation and marginal folds. This was because the PC, as a strong and tough polymer, possessed high crack initiation energy and low crack growth energy. In the impact test, a large amount of energy was absorbed through shear yield deformation, and cracks developed radially from the notch [37]. Compared to pure PC, the shear deformation of the PC-PBAT10 (Figure 9b,e) was relatively weak, and no wrinkles appeared at the edges. This indicated that the number of cracks generated by shear yielding was reduced, resulting in a significant decline in toughness. The impact morphology of the PC-PBAT30 was further changed, as shown in Figure 9c,f. It can be clearly seen that the large shear deformation disappeared and was replaced by relatively flat and small wrinkles that were unable to absorb impact energy, exhibiting poor toughness.

### 3.5. The 3D Printing Performance of the PC/PBAT Blends

The introduction of the PBAT component improved the processing performance of the PC, making the PC/PBAT blends processable at 250 °C, which was a prerequisite for the application of the PC/PBAT blends in MEX technology. In addition to suitable processing temperature, the MEX technology also requires printing materials to possess certain melt fluidity, reasonable softening temperature, good dimensional stability, etc. [38]. Therefore, whether the material can be used for MEX needs to be verified through a series of experiments.

The PC/PBAT blends were prepared into uniform filaments with a diameter of approximately 1.75 mm by the twin-screw extruder, and then long strip specimens were fabricated using the MEX 3D printer (S1 Architect 3D, Guoguang Instruments, Shanghai, China). The printability of the PC/PBAT blends was shown in Table 9. It was found that although pure PC could be smoothly extruded into fine filament, molten PC was quickly cooled and solidified after being extruded from the nozzle, making it unable to adhere to the platform. This was because the T_g_ of pure PC reached 147 °C, which was much higher than the upper temperature limit of the printer platform (110 °C). The situation of the PC-PBAT10 blend was similar to that of pure PC. As expected, since the T_g_ of the PC-PBAT20 was lower than 110 °C, the extruded filaments were softened at this platform temperature and were able to adhere to the printing platform successfully. It can also be seen from Table 9 that the 3D printed specimen based on the PC-PBAT20 showed good dimensional stability and low warping degree. As the PBAT content further increased, the T_g_ of the PC/PBAT blend continued to decrease, and the requirement for platform temperature also decreased. In other words, as long as the platform temperature was still higher than the T_g_ of the blend, the extruded filaments could maintain adhesion while reducing the energy consumption of the printing equipment. Therefore, both the PC-PBAT30 and PC-PBAT40 blends possessed favorable 3D printing performance.

### 3.6. Effect of MBS on Properties of PC/PBAT Blends

#### 3.6.1. Mechanical Properties

From the above studies, we learned that the toughness of the PC/PBAT blends decreased sharply due to the inability to absorb impact energy effectively. Methyl methacrylate-butadiene-styrene terpolymer (MBS), as a typical core-shell toughening agent, may have a good toughening effect on the PC/PBAT blends. Considering the poor heat resistance and low softening temperature of the PC/PBAT blends with high PBAT contents, the influence of the MBS on mechanical properties of the PC/PBAT blends was investigated using the PC-PBAT30 as polymer matrix.

**Figure 10:**
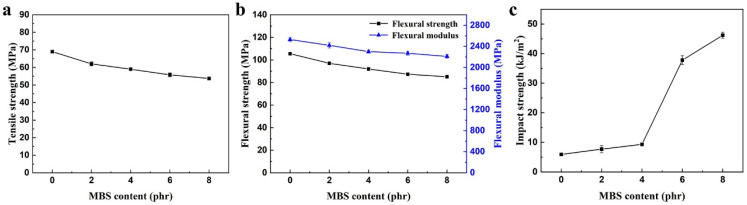
Mechanical properties of the PC/PBAT/MBS blends with different MBS contents: (**a**) tensile strength, (**b**) flexural strength and flexural modulus, (**c**) impact strength.

The mechanical properties of the PC/PBAT/MBS blends with different MBS contents are shown in Figure 10. Due to the fact that the MBS is a thermoplastic elastomer, the strength and modulus of the PC/PBAT/MBS blends showed a slight decrease. Compared to the PC-PBAT30, the tensile strength retention rate of the PC-PBAT30/MBS-6 was 80.2%, while the flexural strength and modulus retention rates were 82.8% and 89.5%, respectively. Therefore, the PC/PBAT/MBS blend system still maintained a certain strength and modulus. Regarding the impact strength, the MBS had a significant toughening effect on the PC/PBAT blends as expected. It was noteworthy that the impact strength of the PC-PBAT30/MBS-6 blend underwent a sudden change, reaching 37.8 kJ/m^2^, which was 6.38 times that of the PC-PBAT30. This indicated that the brittle–toughness transition occurred when the MBS content reached 6 phr. In conclusion, the MBS was an effective toughening agent for the PC/PBAT blend system.

#### 3.6.2. Morphology

**Figure 11:**
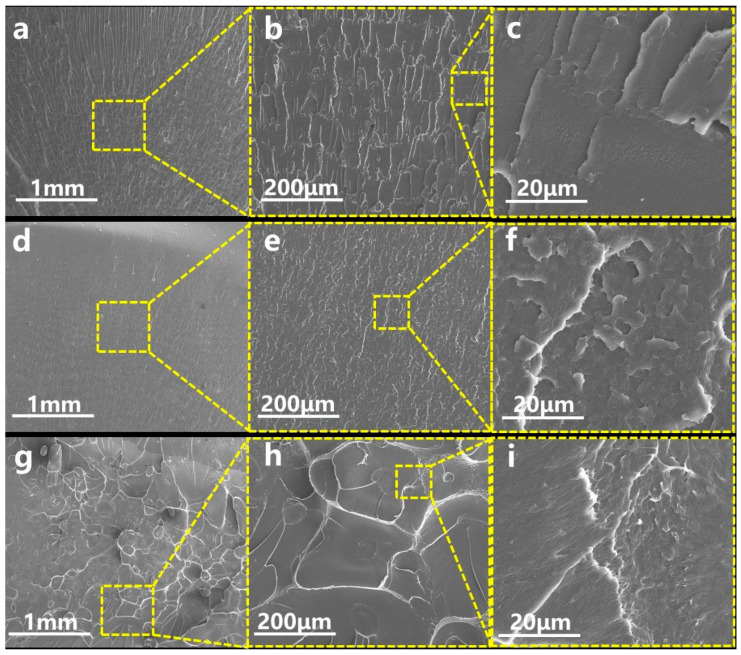
Impact-fractured surface morphology of the PC/PBAT/MBS blends: (**a**–**c**) PC-PBAT30, (**d**–**f**) PC-PBAT30/MBS-4, (**g**–**i**) PC-PBAT30/MBS-6.

In order to clarify the toughening mechanism of the MBS, the impact-fractured surface morphologies of the PC-PBAT/MBS blends before and after the brittle–toughness transition were observed, as shown in Figure 11. It was found that the surface of the PC-PBAT30 without the MBS was flat (Figure 11a–c) and there was no apparent local shear deformation, so impact stress could not be effectively transferred and absorbed by the polymer matrix. After adding 4 phr of the MBS, the surface of the blend remained smooth, but more microcracks appeared (Figure 11d–f). Notably, the impact-fractured surface of the PC-PBAT30/MBS-6 produced apparent changes with numerous grid-like cracks observed on the surface (Figure 11g–i), indicating that the MBS played a stress concentration role, thereby triggering shear deformation around it [39]. When the MBS content (≤4 phr) was too low, the distance between MBS particles was too far to transfer stress, and impact energy was only absorbed by the deformation of the MBS itself. This deformation was extremely limited and could not be transmitted to the matrix, so the enhancement effect on the impact strength was not distinct in this case. When the MBS content exceeded a certain threshold (>4 phr), the spacing between MBS particles was shortened to form interconnections, thus constructing a stress transfer network and effectively preventing crack propagation [40]. In fact, the significant improvement in toughness of the PC/PBAT/MBS blend system was not only due to the MBS acting as stress concentration points, but also due to good compatibility between the continuous PC/PBAT matrix and the MBS component. Therefore, the blend system showed favorable comprehensive mechanical properties.

#### 3.6.3. The 3D Printing Performance

The PC/PBAT blend system had good 3D printing performance, but the toughness of this blend system was poor, which could not meet the needs of products in practical applications. The MBS was proven to be an effective toughening agent for the PC/PBAT blend system. Considering the mechanical properties, heat resistance, and other aspects, the influence of the MBS on the 3D printing performance of the PC/PBAT blend system was studied using the PC-PBAT30 blend as polymer matrix, as shown in Table 10. It was found that all the blends were printed smoothly, and corresponding 3D printed products exhibited excellent dimensional stability without warping. The incorporation of the MBS had little effect on the printing performance of the blend, and the PC/PBAT/MBS blend system also showed favorable printability.

Currently, most desktop-level 3D printers cannot reach a working temperature of 300 °C, and commonly used MEX printing materials are only limited to low melting point polymers such as PLA and ABS. With the help of simple polymer blending technology, the PC/PBAT blend system prepared in this work successfully overcame the application difficulty of high melting point engineering plastic PC in desktop 3D printing equipment. The excellent compatibility between the PC and PBAT components was crucial for improving the 3D printing performance of the PC. The introduction of the PBAT components not only significantly improved the processing performance of the PC, but also reduced the T_g_ of the PC, which was a necessary condition for completing the MEX 3D printing. To adapt to the harsh usage environment, an efficient toughening agent was introduced into the PC/PBAT blends, resulting in a PC/PBAT/MBS blend system with favorable mechanical properties and 3D printing performance. This work was of great significance in expanding the types of MEX printable materials and improving MEX printing performance. On this basis, studying the impact of 3D printing parameters on related properties of 3D printed products and achieving the functionalization of MEX printable materials are two important research directions in the future.

## 4. Conclusions

The introduction of the PBAT significantly improved processing flowability of the PC, making PC/PBAT blends processable at 250 °C. The DSC and DMA results indicated that the PC and PBAT components were completely compatible by comparing the glass transition temperature. Regardless of the PBAT content, the blend system had only one T_g_, and the T_g_ value decreased with increasing PBAT content, which basically conformed to fitting curve of the FOX equation. It was also observed from SEM images that the PC/PBAT blend system exhibited a single continuous phase structure. In terms of mechanical properties, the toughness of the PC/PBAT blend system decreased dramatically despite maintaining high mechanical strength. The MBS was proved to be an effective toughening agent for the PC/PBAT blends. The impact strength of the PC-PBAT30/MBS-6 reached 37.8 kJ/m^2^, which was 6.38 times that of the PC-PBAT30. The impact-fractured surface morphology demonstrated that the brittle–toughness transition occurred in the PC/PBAT/MBS blend system when the MBS content reached 6 phr. This was due to the fact that the MBS particles caused shear yield deformation in the polymer matrix and greatly absorbed impact energy. Regarding 3D printing process, since the T_g_ of pure PC far exceeded the upper limit temperature of the printing platform, pure PC could not adhere to the printing platform normally. When the PBAT content was more than 20%, the PC/PBAT blends were smoothly printed, and corresponding 3D printed products showed good dimensional stability. Moreover, the MBS had little effect on the 3D printing performance. In conclusion, the PC/PBAT/MBS blend system possessed favorable comprehensive mechanical properties and 3D printing performance. This work expanded the types of MEX printable materials through facile polymer blending technology and provided important guidance for developing new MEX materials.

## Figures and Tables

**Table 1:** Designation and detailed formulations of PC/PBTA blends.

Sample	PC (%)	PBAT (%)
PC	100	0
PC-PBAT10	90	10
PC-PBAT20	80	20
PC-PBAT30	70	30
PC-PBAT40	60	40

**Table 2:** Designation and detailed formulations of PC/PBTA/MBS blends.

Sample	Matrix (100 phr)		MBS (phr)
	PC (%)	PBAT (%)	
PC-PBAT30/MBS-2	70	30	2
PC-PBAT30/MBS-4	70	30	4
PC-PBAT30/MBS-6	70	30	6
PC-PBAT30/MBS-8	70	30	8

**Table 3:** The 3D printing parameters used in the sample preparation.

Parameter	Nozzle Diameter	Nozzle Temperature	Printing Speed	Layer Thickness	Raster Angle	Infill Density	Platform Temperature
Value	0.5 mm	250 °C	20 mm/s	0.1 mm	45°/−45°	100%	90 °C

**Table 4:** Equilibrium torque and maximum torque of the PC/PBAT blends.

PBAT Content (%)	0	10	20	30	40
Equilibrium torque	11.7	7.2	4.9	3.5	2.2
Maximum torque	35.8	33.3	21.9	16.1	11.1

**Table 5:** DSC data derived from the DSC curves.

Sample	T_g_ (°C)	T_g_* (°C)
PC	147.0	147.0
PC-PBAT10	121.1	121.9
PC-PBAT20	107.9	97.2
PC-PBAT30	82.1	75.5
PC-PBAT40	59.1	56.2
PBAT	–26.3	–26.3

T_g_*: T_g_ predicted by the FOX equation.

**Table 6:** DMA data derived from the DMA curves.

Sample	Storage Modulus at 25 °C (MPa)	Tan δ Peak Temperature (°C)
PC	1795	170.0
PC-PBAT10	2286	163.6
PC-PBAT20	2382	139.8
PC-PBAT30	2471	114.2
PC-PBAT40	2498	97.7
PBAT	110	−9.4

**Table 7:** TGA data of the PC/PBAT blends.

Sample	T_onset_ (°C)	T_max1_ (°C)	T_max2_ (°C)	Residue (%)
PC	486.7	-	533.6	23.9
PC-PBAT10	403.8	418.5	495.6	19.5
PC-PBAT20	386.5	419.4	475.7	12.8
PC-PBAT30	384.6	414.1	512.7	9.6
PC-PBAT40	377.1	405.1	509.3	7.5
PBAT	371.2	417.6	-	5.4

**Table 8:** VST values of the PC/PBAT blends with different PBAT contents.

PBAT Content (%)	0	10	20	30	40
VST (°C)	148.2	116.3	95.9	78.5	67.0

**Table 9:** The printability of the PC/PBAT blends.

Sample	Platform Temperature (°C)	T_g_ (°C)	Adhere to the Platform	Warping Degree	Image of the 3D Printed Specimen
PC	110	147.0	No	Unable to print	None
PC-PBAT10	110	121.1	No	Unable to print	None
PC-PBAT20	110	107.9	Yes	Very low	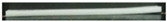
PC-PBAT30	90	82.1	Yes	Very low	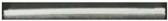
PC-PBAT40	70	59.1	Yes	Very low	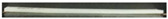

**Table 10:** The printability of the PC/PBAT/MBS blends.

Sample	Adhere to the Platform	Warping Degree	Image of the 3D Printed Specimen
PC-PBAT30	Yes	Very low	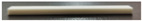
PC-PBAT30/MBS-2	Yes	Very low	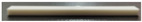
PC-PBAT30/MBS-4	Yes	Very low	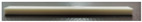
PC-PBAT30/MBS-6	Yes	Very low	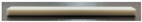
PC-PBAT30/MBS-8	Yes	Very low	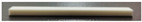

## Data Availability

The data presented in this study are available on request from the corresponding author.

## Supplementary Materials

The following supporting information can be downloaded at: https://www.mdpi.com/article/10.3390/polym15204066/s1, Figure S1: Tg values of the PC/PBAT blends and fitting curve according to the FOX equation; Figure S2: Digital photographs of the pelletized PC/PBAT blends.
