# Supplementary material for: Preparation and Characterization of Polycarbonate-Based Blend System with Favorable Mechanical Properties and 3D Printing Performance
Source: Polymers (Basel). 2023 Oct 12;15(20):4066. doi: 10.3390/polym15204066 (PMC10610018; doi:10.3390/polym15204066)
Supplement: Supplementary file 1 [file polymers-15-04066-s001.zip › polymers-2610939-supplementary.pdf]

Supporting Information

Preparation and characterization of polycarbonate-based blend system with favorable mechanical properties and 3D printing performance

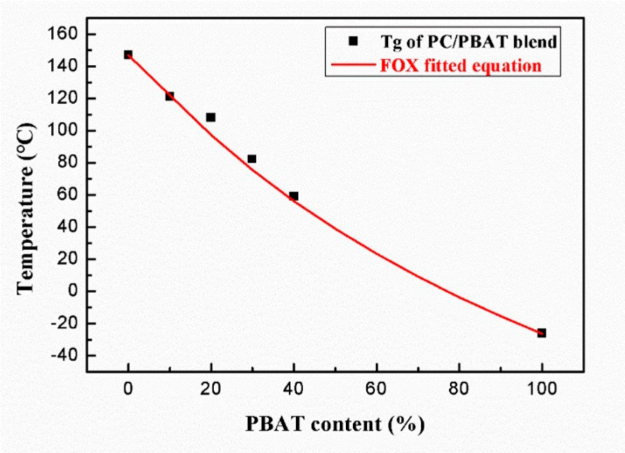

Figure S1. T_g values of the PC/PBAT blends and fitting curve according to the FOX equation

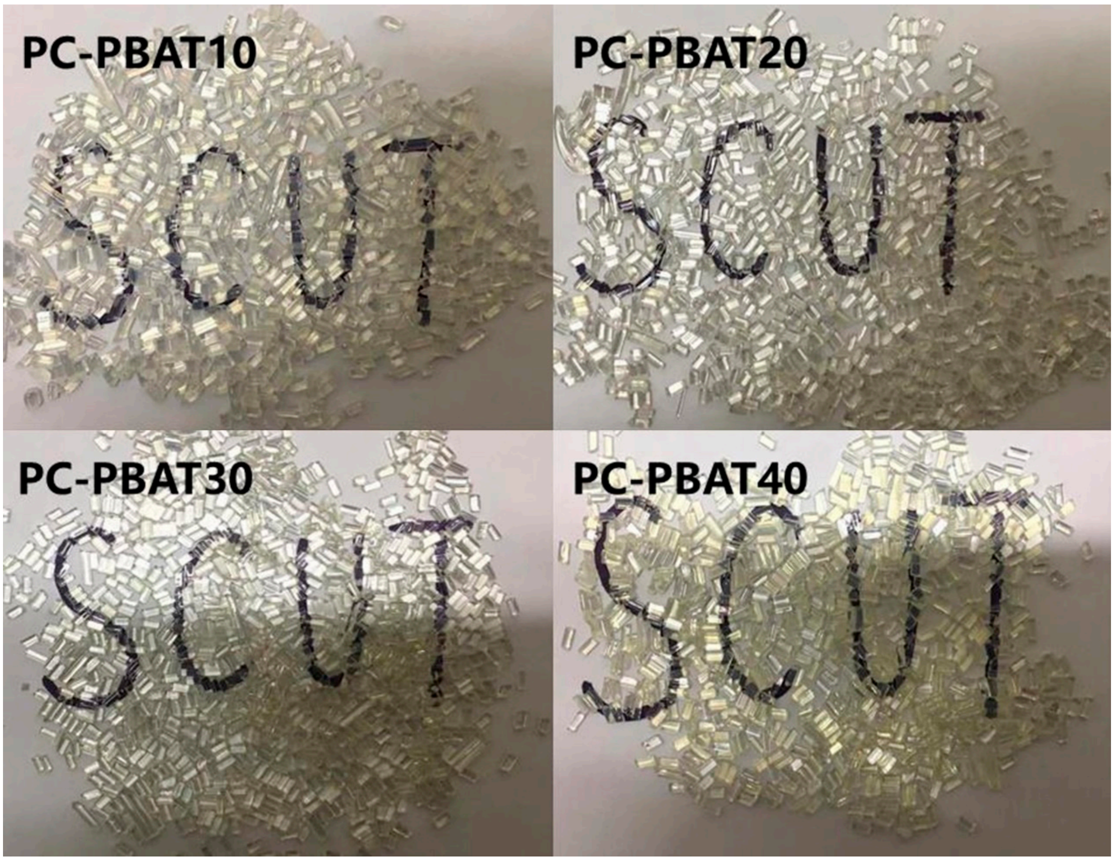

Figure S2. Digital photographs of the pelletized PC/PBAT blends
